# Nurse Mentoring: A Scoping Review

**DOI:** 10.3390/healthcare11162302

**Published:** 2023-08-15

**Authors:** Inmaculada Mínguez Moreno, Delia González de la Cuesta, María Jesús Barrado Narvión, Marta Arnaldos Esteban, Mar González Cantalejo

**Affiliations:** 1 University Hospital Miguel Servet, Institute for Health Research Aragón, 50009 Zaragoza, Spain; iminguez@salud.aragon.es (I.M.M.); marnaldos@salud.aragon.es (M.A.E.); 2 Faculty of Nursing, University of Zaragoza, 50005 Zaragoza, Spain; mjbarrado@salud.aragon.es; 3 Royo Villanova Hospital, Institute for Health Research Aragón, 50009 Zaragoza, Spain; 4 Medical Information, Medical Library, University Hospital Miguel Servet, Institute for Health Research Aragón, 50009 Zaragoza, Spain; mgonzalezc@salud.aragon.es

**Keywords:** mentor, mentee, mentors, mentoring, nursing staff, nurse staff, nurse clinician, clinical nurse, nurse specialist

## Abstract

Introduction: Mentoring programs minimize stress and anxiety in recent graduates and in newly recruited nurses, guiding their careers and enabling them to retain their skills and correctly care for patients. The objective of this scoping review is to explore and summarize the existing literature on mentoring models and programs in the clinical nursing context. Methods: The databases searched include PubMed, Embase, Cochrane Library, Epistemonikos, Cuiden, Scielo, MEDES, OpenGrey, Trove and MedNar. Published and unpublished studies worldwide that included nurse mentoring programs in a clinical context, in public and private systems and primary, secondary and tertiary healthcare settings, and articles published in English, French, Spanish and Portuguese, were included. Nurse students and training specialists were excluded. The papers were screened by two independent reviewers. In cases of discrepancy, a third reviewer made the decision. Results: Eleven studies were included. Most of them were conducted in the USA. A wide range of nurse mentoring programs were identified with highly variable characteristics. The duration of the programs and the evaluation systems were different, but the expected results matched. Conclusions: Mentoring programs need more in-depth and extensive study. In spite of their differences, they all lead to improvements for nurses, patients and organizations. A gender influence was found in our results, which could be studied in future research.

## 1. Introduction

Mentoring is defined as “Intentional mentoring relationships within the setting of a structured program that is bound by a time frame and defined objectives” [1]. It refers not only to the improvement of knowledge, but also the concept of integration within a group and an institution. There exist different forms of mentoring, but not all are the same. It may be called mentoring, a training program, or an induction program. This scoping review focuses only on nurse mentoring programs. 

The first reference to mentoring in the literature was in Greece, but the first reference to specifically nurse mentoring was in England in 1960 [2]. When discussing mentoring, it is important define two essential categories: the mentor and mentee.

A mentor is a professional with important experience and knowledge who assumes the responsibility of guiding, advising, teaching and helping others to learn competency, improve their professional expertise and favor leadership. These attributes are not only about labor requirements, are about personal requirements too. Mentees’ growth requires mentors to improve their trust; motivate them; and use knowledge and reflection (as important tools of learning) to teach them important strategies to resolve problems, make decisions and develop good organization capacity, always in a continuous process of evaluation, while remaining unprejudiced and being a supportive facilitator and partner. 

The mentee is the person who receives the support to be able to develop their skills, as well as to achieve integration in a group or an institution. Assistance quality and security are actually important points in the context of sanitation. Mentoring programs are used in nursing practice as an essential part of the curriculum. This is completely normalized in the student context, and in certain countries in the context of sanitation, especially English ones. 

Actual clinical nursing settings are complex and changing, making them a very interesting candidate for improvement in a clinical context. Nurse mentoring programs provide minimal opportunities for professionals who join new health services, ranging from new graduates to professional nurses who come from other services. The form, duration, organization, and even the impacts of mentoring programs in the literature are very diverse [3]. This review will contribute to determining which nurse mentoring models/processes exist in the clinical context, as well as their impacts and contributions to institutions and to professionals. 

Mentorship, mentoring, training programs, and induction programs have been shown to be fundamental to guaranteeing adaptation and training at the healthcare level of new nurses [4], promoting quality care and integrating theoretical and practical nursing training. Nurse mentoring contributes to reducing the risk of mistakes and professional stress, and has already been shown to reduce the risks of clinical variability and staff mobility, as well as improving satisfaction [5,6].

The objective of this scoping review is to explore and summarize the existing literature on mentoring models and programs in the clinical nursing context, including their forms, duration, organization, and impacts on nursing professionals and healthcare institutions.

Review question(s):

- What kinds of mentoring model or processes exist in the clinic nursing context?
- What is the impact of mentoring programs on nursing professionals?
- What is the impact of nurse mentoring programs on healthcare institutions?

## 2. Materials and Methods

### 2.1. Inclusion Criteria

#### 2.1.1. Participants

This review included studies conducted worldwide that focused on mentoring programs directed specifically at nurses, ranging from bachelor-level nurses to those with doctorate degrees, in a clinical context. The studies primarily focused on nurse students, and academic experts or individuals other than bachelor-level nurses were excluded, with the objective of determining the impact of mentoring programs on people who have at least a bachelor ‘s degree.

#### 2.1.2. Concept

This review considered studies in which the outcomes were related to mentoring programs for bachelor nursing in any clinical context in any country, as well as their impact on nursing professionals, and the impact of apply them in health institutions. Examples of regulatory activities included programs that explained the different methods used, the positive or negative impacts on nursing, as well as the benefits or drawbacks for institutions, like promoting quality care and integrating theoretical and practical nursing training and reducing the risk of mistakes and professional stress, having already been shown to reduce the risks of clinical variability and staff mobility, as well as improving satisfaction.

#### 2.1.3. Context

This review encompassed mentoring programs in various clinical areas within public and private healthcare systems. Primary (primary care providers), secondary (specialists) and tertiary (specialized care in a hospital) settings were included. The countries selected for inclusion in this scoping review were limited to English-speaking, Spanish-speaking, French-speaking and Portuguese-speaking countries. English was selected because 95% of publications are written in this language. The rest of the languages were selected based on the knowledge of the authors.

#### 2.1.4. Types of Sources

This scoping review considered all of the information included in any of the databases and the grey literature for unpublished documents, trying our best to capture reality in the most reliable way. The reviewers decided to keep the source of information “open” to allow for the inclusion of any and all types of studies. Otherwise, important information could be lost.

### 2.2. Methods

The objectives, inclusion criteria and methods for this scoping review were specified in advance and documented in a protocol, registered on the Open Science Framework platform (http://osf.io/uh7q2?view_only=3c7eec1ccbed4798a7e4223472d551c2 (accessed on 1 March 2021)). The Joanna Briggs Institute (JBI) methodology for scoping reviews was followed [6].

#### 2.2.1. Search Strategy

The search strategies were developed by an experienced medical librarian (MGC) and peer reviewed by another librarian (CCA) according to best practice recommendations [7]. The search strategies used Boolean operators, truncation terms and keywords, identified using medical subject headings (MESH, via the US National Library for Medicine MESH browser), and Embase subject headings (EMTREE, via Embase.com (accessed on May 2020)). The reproducible search strategy for PubMed can be found in Table 1.

To identify potentially relevant documents, a comprehensive literature search was conducted using the most impactful bibliographic databases: MEDLINE (PubMed), Embase (via Embase.com (accessed on May 2020)) and Cochrane Library. We also searched in additional databases, including Epistemonikos, Cuiden (Index Foundation), Scielo (Scientific Electronic Library Online) and MEDES (Medicine in Spanish), as well as in some grey literature databases for unpublished documents: OpenGrey, Trove and MedNar. Finally, we used the search engine Google Scholar. All searches were run in May 2020. The analysis time was arduous, which prolonged the investigation, ending in October 2022. No time limits were applied to the search strategy. The studies were limited to those published in English, French, Spanish and Portuguese.

#### 2.2.2. Source of Evidence Screening and Selection

The final search results were exported into Mendeley, which was used to manage the references and remove duplicates. The Rayyan [8] citation management platform was used to screen and review the articles. Rayyan is largely intuitive to use, with significant potential to lighten the load of review authors by speeding up this part of the process. Titles and abstracts were screened by two independent reviewers for assessment against the inclusion criteria for the review. Any disagreements that arose between the reviewers were resolved by a third reviewer guided by the inclusion criteria. The full texts of potentially relevant evidence were retrieved for further review against the inclusion criteria.

#### 2.2.3. Data Extraction

The data extraction process followed a predefined data extraction form based on the review protocol. Data were extracted from papers included in the scoping review by three independent reviewers. There was no need to contact the authors of the papers for missing or additional data. Any disagreements that arose between the reviewers were resolved through discussion and consensus.

#### 2.2.4. Analysis and Presentation of Results

A data extraction table based on recommendations from the *JBI Reviewer’s Manual* [6] and specified in the review protocol, registered in the Open Science Framework platform, was used. The information extracted included standard information (title, author, year of publication, language, country of origin and study design).

During data extraction, it was found that the information related to the characteristics of the nurse mentoring programs was highly variable. For this reason, a second table was created to facilitate the description of the mentoring model/processes that exist in the clinic nursing context. The information added included the hospital’s name and features, program, duration, participants, objectives, development, outcomes and measurement tools.

## 3. Results

### 3.1. Search Results

**Figure 1:**
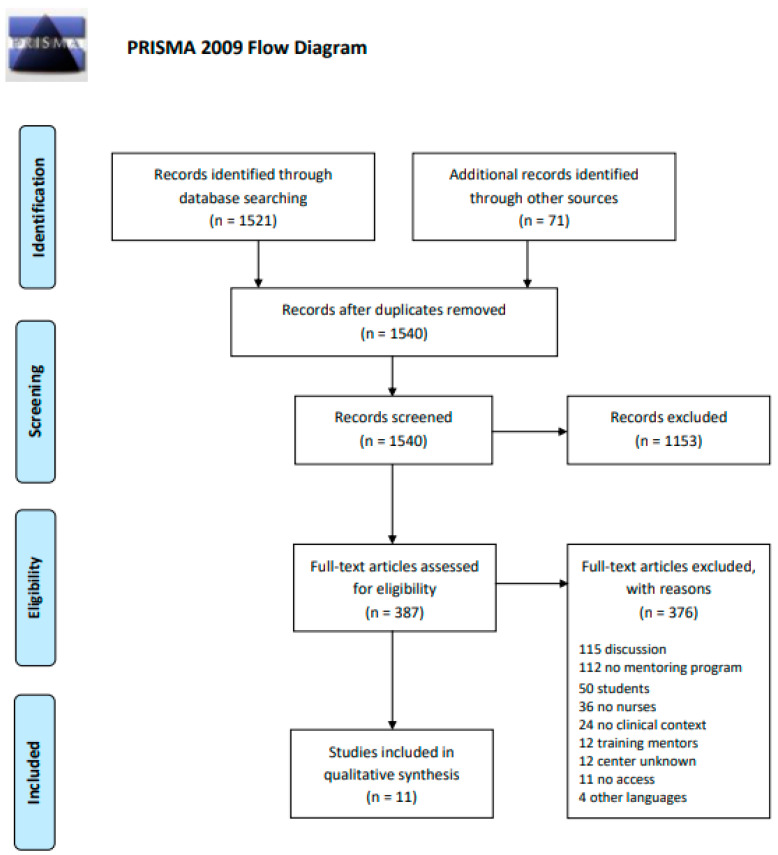
Prisma Flow Diagram.

The database searches identified 1521 records. In total, 71 additional records were identified through other sources. A total of 52 duplicates were removed, leaving 1540 records. A further 1153 were not relevant, as determined by the initial screening of titles and abstracts. A total of 387 articles were retrieved in full text. Of these, 376 were excluded. A total of 11 records were included in this review. The results of the search are presented in a Preferred Reporting Items for Systematic Reviews and Metanalyses extension for Scoping Reviews (PRISMAScR) flow diagram [9] (Figure 1).

### 3.2. Inclusion of Sources of Evidence

**Figure 2:**
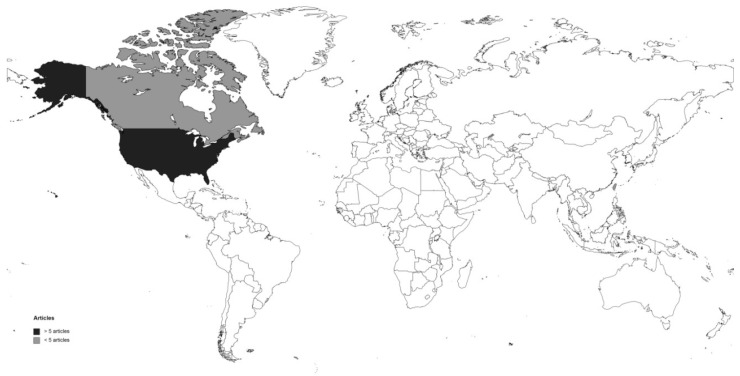
Heat map of the literature. This map illustrates the articles’ countries of origin. Lighter color represents smaller numbers of articles and darker color represent greater numbers of articles.

Eleven articles met the inclusion criteria and were included in this review. The articles included nine journals, eight of which were nursing journals [10,11,12,13,14,15,16,17,18]. The publication dates ranged from 1992 to 2021. Of the 11 included articles, only one was published prior to 2006 [10] and most of them were published later, and we did not find any differences between the 2000s and the 2010s in terms of the number of publications. All of the articles were written in English. The articles were categorized by country of origin; most of them were conducted in the USA [10,11,12,13,16,17,18,19], one in Canada [14] and one in Taiwan [19]. The countries of origin of the included papers are shown in Figure 2.

Descriptive studies were the most common research design identified [4,10,11,12,13,16,17,18]. Further details of the characteristics of the included articles are shown in Table 2.

A wide range of nurse mentoring programs were identified with highly variable characteristics. Most of them were developed in third-level hospitals, and only one of the studies reported data from long-term-care facilities and community organizations [14]. Only the program in Taiwan was developed in a public hospital [19]; the rest were developed privately. Regarding the types of programs, there were programs created specifically for the studies. “Norton Navigators” [11] and “Nurses Nurturing Nurses” [13] are structured programs that were widely named in the literature on this subject and were recognized by researchers throughout the selection process of the articles. The duration of the programs was very diverse, from eight weeks [16,18] to eighteen months [11], as was the number of members in the groups created. Only one of the studies included the same number of mentors as the number of mentees [18]^.^ A few programs were developed with external support from sources such as universities or nursing associations [13,17].

All mentoring programs were orientated toward new nurses, with few/no programs with different objectives, and ranged from improving job environments and increasing retention to improving the evidence based on practice or determining the satisfaction and organizational commitment of new nurses. One study used a mentoring support to facilitate evidence-based practice [15]. Regarding the development of the programs, this was the section in which we found more variability, but all of the programs agreed on a promotion period, the pairing of recent nurses with experience nurses, and a final evaluation.

The results of the programs were varied, as were their objectives. Regarding the impact on nursing professionals, seven studies talked about the enhancement of nursing [4,10,13,14,15,18,19]. Regarding impact on healthcare institutions, at least seven studies pointed to the improvement of the retention of nurses [4,11,12,13,16,17,18], two of the studies talked about economic savings [4,11] and only one talked about higher patient satisfaction [11]. In addition, one more talked about the learning and application of and challenges and barriers to succeeding in implementing evidence-based practice [15].

The measurement tools were all different. Further details of the characteristics of the nurse mentoring programs are shown in Table 3.

## 4. Discussion

Mentoring programs are frequently used to retain professionals in centers or in zones with difficult access or with problems with generational relief, as is the case in New Zeeland, where it is practically required to perform programs that allow for fast adaptation and immersion in the practice, to ensure that there are problems with having nurses in theses contexts.

Generalist and specialist nurses benefit from these programs, because they allow them to grow quickly in all of the situations [20]. In addition, in special situations such as the pandemic, mentoring programs have allowed for the training of new nurses, decreasing pressure, improving security and increasing retention in institutions [17].

Mentoring programs in large hospitals contribute to increased nurse allegiance and reduce mobility and turnover, making these hospitals more attractive to new professionals [21,22]. These programs allow for internal and sustainable changes in organizational culture, which is very complicated, especially in big hospitals [23]. In addition, manager nurses would benefit from these kinds of programs both in terms of the incorporation of new elements in the management of teams and for organization as a whole [24]. Regarding gender, in this field, the female binomial is the most representative, where most mentors and mentees are women. There have been observed differences with the masculine approach, except in promotion in the workplace. The feminine approach improves clinical skills but does not require competitiveness for professional development or promotion within the field.

Mentoring programs have the biggest influence on clinical professionals, who see increases in their professional growth and more security in their own abilities [23], as well as new nurses [25]. However, nurses who are going to be mentors also need to develop abilities and strategies to effectively communicate their knowledge and experience to mentees, with a manner of professionalism and trust that does not lead to confusion about their roles.

## 5. Conclusions

There are important heterogeneity in all of the studies’ aspects, including the program duration, number of participants and objectives, as well as the structures and evaluations tools. These results suggest that mentoring programs need more in-depth and extensive studies. In spite of their differences, they all lead to improvements for nurses, patients and/or organizations.

All mentoring programs aim to support the transition of graduate or experienced nurses to new service areas. Mentoring programs improve practice and retention, enhance nurse performance and create a supportive and positive work environment.

The impact on institutions’ organization is positive, decreasing mobility, leading to references and making hospitals attractive to new professionals, enabling new leaders to be found in the organizations and promoting professional development. All of these factors are creating a global and lasting change in organizational culture, which is especially complicated in large hospitals.

The feminine approach improves clinical skills but does not require competition for development or promotion within the profession. This finding suggests that it would be interesting to study or contemplate, in future studies, a gender perspective and its impact in mentoring.

## Figures and Tables

**Table 1:** Reproducible search strategy, with an update in October 2022.

Search	Query
#1	Mentor*[tiab] OR Mentee*[tiab]
#2	Mentors[Mesh] OR Mentoring[Mesh]
#3	#1 or #2
#4	Nursing Staff*[tiab] OR Nurse staff*[tiab] OR Nurse Clinician*[tiab] OR Clinical Nurs*[tiab] OR Nurse Specialist[tiab]
#5	“Nursing Staff”[Mesh] OR “Nurse Specialists”[Mesh] OR “Nurse Clinicians”[Mesh]
#6	#4 OR #5
#7	#6 AND #3

**Table 2:** Characteristics of included articles.

Code	Author/Title	Year of Publication	Country	Study Design	Reference
S01	Brito, H.H. Nurses in Action. An innovative approach to mentoring. *J. Nurs. Adm.* 1992, *22*, 23–28.	1992	USA	Descriptive study	[10]
S02	Zucker, B.; Goss, C.; Williams, D.; Bloodworth, L.; Lynn, M.; Denker, A.; Gibbs, J.D. Nursing Retention in the Era of a Nursing Shortage. *J. Nurses Staff Dev.* 2006, *22*, 302–306.	2006	USA	Descriptive study	[11]
S03	Latham, C.L.; Hogan, M.; Ringl, K. Nurses supporting nurses: Creating a mentoring program for staff nurses to improve the workforce environment. *Nurs. Adm. Q.* 2008, *32*, 27–39.	2008	USA	Descriptive study	[4]
S04	Persaud, D. Mentoring the New Graduate Perioperative Nurse: A Valuable Retention Strategy. *AORN J.* 2008, *87*, 1173–1179.	2008	USA	Descriptive study	[12]
S05	Grindel, C.G.; Hagerstrom, G. Nurses nurturing nurses: Outcomes and lessons learned. *Medsurg Nurs.* 2009, *18*, 183–187, 194	2009	USA	Descriptive study	[13]
S06	Weng, R.-H.; Huang, C.-Y.; Tsai, W.-C.; Chang, L.-Y.; Lin, S.-E.; Lee, M.-Y. Exploring the impact of mentoring functions on job satisfaction and organizational commitment of new staff nurses. *BMC Health Serv. Res.* 2010, *10*, 240.	2010	Taiwan	Qualitative study	[19]
S07	Hunsberger, M.; Baumann, A.; Crea-Arsenio, M. The road to providing quality care: Orientation and mentorship for new graduate nurses. *Can. J. Nurs. Res.* 2013, *45*, 72–87.	2013	Canada	Qualitative study	[14]
S08	Friesen, M.A.; Brady, J.M.; Milligan, R.; Christensen, P. Findings from a Pilot Study: Bringing Evidence-Based Practice to the Bedside. *Worldviews Evid.-Based Nurs.* 2017, *14*, 22–34.	2017	USA	Pilot study	[15]
S09	Mijares, A.H.; Radovich, P. Structured Mentorship and the Nursing Clinical Ladder. *Clin. Nurse Spec.* 2020, *34*, 276–281.	2020	USA	Descriptive study	[16]
S10	Krofft, K.; Stuart, W. Implementing a Mentorship Program for New Nurses During a Pandemic. *Nurs. Adm. Q.* 2021, *45*, 152–158.	2021	USA	Descriptive study	[17]
S11	Gayrama-Borines, Z.; Coffman, S. Effectiveness of Mentorship Program in the Emergency Department. *J. Nurses Prof. Dev.* 2021, *37*, 107–111.	2021	USA	Qualitative study	[18]

**Table 3:** Characteristics of the nurse mentoring programs.

Code	Hospital’s Name and Features	Program/Duration	Participants	Objectives	Development	Outcomes	Measurement Tools
S01	Jackson Memorial Hospital Second largest medical center in the United States, with more than 1500 beds and 2000 nurses. Miami, Florida EEUU Private	“Nurses in action”/6–12 months	70 advisors 111 advisees	Increase the pool of qualified applicants for vacant positions. Provide nurses in new roles with advisors to enhance their performance.	The program was promoted via staff meetings and fliers, in nursing orientation, in nursing opportunities listings and in newsletters. A data base was used to generate lists of advisors and advisees that were distributed so that participants could choose potential partners. A luncheon was designed with the purpose of networking and sharing. Monthly half-hour lunches were developed to accommodate the staff nurses´ schedule. Lunch topics have included 10-minute presentation on mentoring, goal-setting, expanded roles of nurses, advisor/advisee success stories, self- assessments and piloting mentor programs at the unit level.	Provide an increased pool of potential successors to fill vacant positions quickly and enhance the selection process for promotions. Enhance the nursing profession through the development of its members.	A questionnaire for participants was sent six months after they began the program
S02	Norton Healthcare 5 hospital system 2500 nurses Louisville, Kentucky EEUU Private	“Norton Navigators”/18 months	95 navigators 260 protégés	Improve retention	Matches recent nursing school graduates with experienced nurses. Mentors receive specialized training, monetary incentives. The program was promoted in new graduates´ interviews, mailing and in the newsletter. They received an acceptance package. There is a program coordinator.	Improve retention rate of new nurses and reduced the overall turnover rate for new graduates. Save money, higher patient satisfaction and an increase in recruitment.	Risk analysis at a 6 months
S03	2 hospital system 850 beds California EEUU Private	“Nurses supporting nurses”	76 mentors 95 mentees	Improve the workforce environment	A steering committee, including the hospital liaison and the university project team was created. Brochures, posters and flyers were created to market the project. Nursing leadership was informed. Recruiting experienced staff nurses to serve as mentors. Application form to be considered for the project. Individual Web pages were given to each mentor and mentee to help the selection and match. Educational sessions were developed. Quarterly mentor support meetings.	Improvement of patient and nurse satisfaction, nurse vacancy and retention rates and patient safety data relating to fall and Pressure Ulcer prevention. Save money.	Decisional Involvement Scale
S04	OSF Saint Anthony Medical Center Rockford, Illinois EEUU Private	“AORN’s Perioperative Nursing Course 101”/16 months	9 mentors 27 mentees	Improve retention	Nurse volunteers were solicited (2 years of experience as a perioperative nurse). Five nurses volunteered to be members of the Mentorship Planning Committee. The Committee members promoted the program. A mentorship program folder was created. They selected a mentor for each mentee and planned the meeting that would bring all of the mentors and mentees together for the first time. The mentor and mentee each were asked to sign a contract. Each mentorship pair was asked to meet on at least a monthly basis.	Improve retention	Evaluation survey
S05	18 hospitals or hospital systems agreed to participate in the project. These hospitals were located around the country: Northeast (n = 4), South (n = 10), North Central (n = 3), West (n = 1). Private	“Nurses Nurturing Nurses”/12 months	107 mentees 119 mentors	Enhance nurses´ job satisfaction Improve retention	The mentorship program was developed by The Academy of Medical-Surgical Nurses in a hospital. Agencies interested purchased a packet of materials. The Academy appointed a program coordinator. Once an agency decided to participate, leaders were asked to appoint a site coordinator to serve as project director, who was responsible for matching the mentor/mentee dyads, providing orientation for the mentors and mentees, facilitating processes within the agency, and assisting in collection of evaluation data.	New nurse confidence increase. A formal mentorship program may be effective in improving nurse retention if the hospital/ hospital system makes a firm commitment to support the mentoring program.	Evaluation materials were collected four times over the 12-month period. Intent to Stay/Job Diagnostic Survey Nurse job satisfaction survey New Nurse Confidence Scale (NNCS) Mentee “Assessment of the Relationship with the Mentor” Mentor “Assessment of the Relationship with the Mentee” Mentee’s Satisfaction with the Program Mentor’s Satisfaction with the Program
S06	3 regional hospitals in Taiwan Public	Mentoring program for new staff nurses/ 2–4 months	306 nurse subjects were obtained	Job satisfaction and organizational commitment of new nurses	All the hospitals that were part of the study have an existing formal program that required mentors to give guidance and assistance to new staff nurses for at least two months. Nursing managers usually assigned senior nurses as mentors to guide the new staff nurses. The types of this program include classes, seminar, workshop, or conference.	Effective mentoring will reinforce the job satisfaction of the new nurses and their commitment to the hospital The psychosocial support and career development appear lower	Employed self- administered questionnaires to collect research data
S07	Hospitals, long- term-care facilities and community organizations Ontario, Canada	“Nursing Graduate Guarantee”/12 weeks	1198 employers	Facilitate the transition of new graduate nurses to professional practice	This study was part of a policy evaluation of The Ontario health ministry conducted annually from 2007 to 2010. HealthForceOntario services were used to e-mail all newly graduated nurses. To participate in the Nursing Graduate Guarantee, employers and New Graduated Nurses had to register on a Web-based employment portal created by HealthForceOntario. Including 3 to 6 days of general orientation; clearly defines the roles of newly graduated nurses, mentors, and orientation leaders; and ensure the use of a learning plan by and New Graduated Nurses and mentors Supervision is generally one-to-one. In some cases, however, the and New Graduated Nurses rotates to a different unit for increased clinical exposure, and thus is supervised by more than one mentor	Mentorship increased the new graduate nurses confidence and allowed them to make clinical decisions in safe, protected environment. The program provided vital support and helped new graduate nurses move from students to practicing nurses.	Focus groups were held with employers (they were contacted by e-mail) and individual interviews were conducted with newly graduated nurses and mentors.
S08	Multihospital system (5 units in 5 hospitals) EEUU Private	Structured evidence- based practice education with mentoring innovation for nurses/ 14–16 weeks	169 registered nurses	Mentoring support to adopt evidence- based practice. Empower nurses to proactively seek the best available evidence, improve patient care and outcomes and diffuse the implementation of EBP in the nursing unit.	Johns Hopkins Nursing Evidence-Based Practice Model´s. The practice question-evidence-translation process provided a concrete structure for evidence- based practice education with mentoring program planning. One team leader and one clinical registered nurse interested in serving as a resource nurse were recruited from each unit. Pre- and post-data were collected from convenience sample of nurses. Resources were allocated including educational material and designated personnel. An educational program was developed. A clinical nurse from each pilot unit serves as mentor to other staff. The initial group didactic session included foundational steps in the process. Classroom-based instruction for the education program was offered. The intervention included a launch. Group sessions for literature review and ranking or rating were offered via conference calls. Then participants from the pilot units synthesized the evidence and made a determination on overall quality of evidence exist to warrant an implementation project.	Positive movement toward evidence-based practice in project participants. Nurses at all levels of practice require mentoring and coaching to foster evidence-based practice sustainment.	Evidence-based practice beliefs scale and implementation scale Focus groups
S09	Adult medical care and adult step-down surgical care unit 900-bed, level 1 trauma, academic hospital Southern California EEUU Private	“The evidence- based practice program” Short term structured mentorship program/ 8–10 weeks	11 mentees (initial pilot study)	Increase work engagement among staff	Participants were recruited after program presentation. Clinical nurse specialist scheduled a face-to-face or telephone meeting. Follow-up emails were sent every 2 weeks for 8 weeks.	Professional development of nursing staff increases job satisfaction and employment retention	Utrecht Work Engagement Scale Munich Evaluation of mentoring Questionnaire
S10	300 beds community hospital Midwestern EEUU Private	Mentoring program for new registered nurses hired into medical- surgical units/12 weeks	15 mentors 20 mentees	Improve retention	Based on the mentoring program of The Academy of Medical-Surgical Nurses published in 2012.	Improve retention	Intent to Stay/Job Diagnostic Survey Nurse job satisfaction survey Mentee’s Satisfaction with the Program Mentor’s Satisfaction with the Program
S11	Large community hospital in the Southwestern EEUU Private	Mentoring program in the Emergency Department t/ 8 weeks	3 mentees 3 mentors	Helped transitioning registered nurses adjust to the role of emergency nurses	The registered nurses were introduced to the emergency department through a 10- week orientation, where they followed their assigned preceptors through the department´s guidelines. After this period, they were paired with seasoned emergency department nurses. The mentoring program consisted of direct observational oversight, weekly meetings between mentors and mentees, frequent visitations with stakeholders and data collection and analyze. During the first week, they worked on the same shift but managed their own patients. The last 2 weeks of the program were intended for electronic mentoring.	The program helped mentees feel more supported and mentors more empowered; it also improved teamwork significantly, created camaraderie, intensified confidence levels, enhanced professionalism and boosts morale across the emergency department. The program positively impacted both retention and job satisfaction.	The Mentoring Competency Assessment The Intent to Stay/Leave Diagnostic Survey

## Data Availability

All data used in this project are included at the manuscript. No new data were created.
